# Diagnosis and management of septal deviation and nasal valve collapse - a survey of Canadian otolaryngologists

**DOI:** 10.1186/s40463-019-0394-z

**Published:** 2019-12-16

**Authors:** Yiqiao Wang, James P. Bonaparte

**Affiliations:** 1 Faculty of Medicine, University of Ottawa, Ottawa, Canada; 2 Department of Otolaryngology – Head and Neck Surgery, Senior Clinical Investigator, The Ottawa Hospital Research Institute, University of Ottawa, Ottawa, Canada

**Keywords:** Septoplasty, Nasal obstruction, Nasal valve collapse, Survey

## Abstract

**Background:**

Management of nasal valve collapse (NVC) in patients with a septal deviation can be challenging. Our objective was to determine the opinions of Canadian Otolaryngologists regarding the diagnosis and management of nasal obstruction in patients with septal deviation and NVC.

**Methods:**

A twenty-question survey was developed for the purpose of our study. Questions were divided into the following areas: diagnosis, management and prognosis. We included all otolaryngologists who were members of the Canadian Society of Otolaryngology.

**Results:**

The response rate to our survey was 18%. The most commonly identified cause of a failed septoplasty was incomplete septoplasty (41.9%), followed by nasal valve collapse (25.6%). The Cottle manoeuvre (62.8%) and visual inspection (39.5%) were noted to be the most important diagnostic tools for external and internal NVC respectively. However, physicians often rely on a variable number of different examinations when making a diagnosis of nasal valve collapse. When evaluating which patients with a septal deviation also required nasal valve surgery, 27.9% of responders believed the current physical examination methods provided a high accuracy, while 55.8% indicated moderate accuracy and 16.3% indicated low accuracy. Compared to other subspecialties in Otolaryngology, Facial Plastic and Reconstruction Surgeons noted higher septoplasty failure rates in patients with co-morbid NVC.

**Conclusions:**

NVC is an important concern for otolaryngologists performing septoplasty. Although most physicians believe that the physical exam provides a moderate effectiveness when predicting who requires a functional rhinoplasty, diagnostic methods used for NVC is varied and inconsistent.

## Introduction

Septal deviation is a common cause of nasal obstruction, present in up to 80% of the general population [1]. However, many cases of septal deviation are asymptomatic, and the degree or severity of deviation has little to no correlation with the degree of obstruction [2, 3]. This paradox creates a diagnostic dilemma for some patients and surgeons. Not all patients, regardless of symptoms demonstrate an improvement as patient satisfaction after septoplasty ranges between 65 to 80% [4].

One potential cause of treatment failure may be misidentification of other comorbid causes of nasal obstruction, specifically nasal valve collapse (NVC) [5, 6]. Concurrent NVC is often viewed as an important feature to identify prior to a septoplasty to prevent need for revision surgery [7, 8]. Clinicians have developed several physical examinations to assess and diagnose NVC [5, 9–11]. However, a consensus statement by the American Academy of Otolaryngology – Head and Neck Surgery (AAO-HNS) states that although many such tests are available, there is no gold standard [10]. Common tests for diagnosis include the Cottle manoeuvre (cheek displaced laterally with the fingers) and the modified Cottle manoeuvre (ear curette used to support the lower lateral cartilage). Bachman’s Maneuver, although less commonly used and often confused with the Modified Cottle Maneuver, involves digital pressure on the tip of the nose, pushing the nose upward in the sagittal plane (ie a pig nose appearance).

Due to these challenges, a better understanding of how otolaryngologists approach septal deviation and NVC will help guide development of guidelines as well as future research into the area. Therefore, the objective of this study was to determine the opinions of Canadian Otolaryngologists regarding the diagnosis and management of nasal obstruction with septal deviation and NVC. Our secondary objective was to evaluate differences between sub-specialists.

## Methods

### Survey

Research Ethics Board approval was obtained through The Ottawa Hospital (Protocol #20160194-01H). Our team constructed a twenty-question survey for our study. The survey was developed initially from personal experience and peer reviewed literature, then pilot tested with members of the University of Ottawa, Department of Otolaryngology. Once completed and revised, feedback from the American Academy of Otolaryngology – Head and Neck Surgery (AAO-HNS) Rhinology committee was provided. Further revisions were then made, and a final survey was approved. The survey was divided into the following areas: diagnosis, management, and prognosis. All questions were mandatory, and additional responses could be added if required.

### Data collection

Eligible participants were otolaryngologists who were members of the Canadian Society of Otolaryngology – Head and Neck Surgery (CSOHNS). Two email invitations were sent to all members in January and April 2017, which included the survey link and a description of the project. The survey closed in August 2017. Consent to participate was implicit on response, and all responses were gathered anonymously. A third party recognized survey website (www.surveymonkey.com) was used for data collection and storage.

### Data analysis

All information was treated confidentially. Data was exported to excel (Microsoft©, 2018) and Minitab 18 (Minitab Inc) for analysis. Survey data that included continuous data was assessed using an ANOVA for normally distributed data and Kruskal-Wallis test for non-normally distributed data. Categorical data was analysed using Chi-square testing. Significance was defined as *p* ≤ 0.05.

## Results

### Demographics

Demographic data is outlined in Table 1. Eighty-six otolaryngologists responded to our survey from a total of 489 invitations (18%). Respondents were General Otolaryngologists, Facial Plastics and Reconstruction Surgeons (FPRS), and Rhinologists, with the majority having less than 10 years of experience. Type of practice was evenly distributed between community, office and hospital.

**Table 1:** Demographics

Variable	Count	% total
Total Respondents	86	100
Speciality		
General	48	55.8
FPRS	18	20.9
Rhinology	20	23.3
Other	0	0.0
Experience (years)		
< 5	30	34.9
5–10	24	27.9
10–15	8	9.3
15–20	4	4.7
> 20	20	23.3
Practice		
Hospital	26	30.2
Office	22	25.6
Community	38	44.2

### Diagnosis

Surgeons utilized up to seven different physical examination or historical findings when identifying internal and external nasal valve collapse (Table 2). Visual inspection was identified as the most common test for the diagnosis of both internal and external NVC. However, the Cottle Manoeuvre was most commonly identified as the most important single test for the diagnosis of external NVC. In terms of internal NVC, both visual inspection and the modified Cottle maneuver we identified as the most important examination at near similar rates (Table 3). 52.2% of all respondents identified visible valve collapse on inspiration as the most important finding that suggested the need for nasal valve repair at time of septoplasty, with rates of 66.7% for General Otolaryngologists and 44.4% for FPRS (Table 4). In contrast, the majority of Rhinologists (37.5%) noted that static narrowing of the nasal valve was the most important finding (Table 4). Furthermore, with respect to diagnostic accuracy (the ability to predict which patients with a septal deviation also required nasal valve surgery), most physicians believed the physical exam provided moderate accuracy. There was a trend towards FPRS indicating a higher level of accuracy compared to other specialists however this did not reach statistical significance (Table 5, *p* = 0.23).

**Table 2:** Methods Used to diagnose Nasal Valve Collapse in a typical clinical encounter

Examination*	Internal		External	
	n	% Total	n	% total
Visual Inspection	80	93.0%	78	90.7%
Cottle Maneuver	48	55.8%	33	38.4%
Modified Cottle Maneouvre	34	39.5%	31	36.0%
Failed Septoplasty	38	44.2%	20	23.3%
Bachman’s	10	11.6%	10	11.6%
Trial of BreathRight	2	2.3%	0	0.0%
Fiber-optic Nasolaryngoscopy	0	0.0%	0	0.0%
Acoustic Rhinometry	2	2.3%	2	2.3%

* responders to this question were allowed to chose all the physical examinations they used in a typical clincal encounter

**Table 3:** List the physical examination method that you rely on the most for the diagnosis of Nasal Valve Collapse

	Internal		External	
	n	% Total	n	% Total
Visual Inspection	34	39.5%	2	2.3%
Cottle Maneuver	22	25.6%	54	62.8%
Modified Cottle Maneouvre	22	25.6%	6	7.0%
Failed Septoplasty	6	7.0%	10	11.6%
Bachman’s	0	0.0%	4	4.7%
General Physical Exam	0	0.0%	6	7.0%
Experience	2	2.3%	0	0.0%
Fiber-optic Nasolaryngoscopy	0	0.0%	0	0.0%
Breathe Right Trial	0	0.0%	0	0.0%
History Alone	0	0.0%	0	0.0%
Other	0	0.0%	4	4.7%

**Table 4:** Most Important finding indicating need for Functional Rhinoplasty

	Total		General		FPRCS		Rhinology	
	n	%	n	%	n	%	n	%
Collapse on Inspiration	42	48.8%	32	66.7%	8	44.4%	2	10.0%
Cottle Maneuver	6	7.0%	4	8.3%	0	0.0%	2	10.0%
Modified Cottle	2	2.3%	2	4.2%	0	0.0%	0	0.0%
Static Narrow Nasal Valve	8	9.3%	0	0.0%	2	11.1%	6	30.0%
Severe Nasal Symptoms	12	14.0%	6	12.5%	2	11.1%	4	20.0%
Location of Septal Deviation	4	4.7%	2	4.2%	2	11.1%	0	0.0%
Intra-op findings	2	2.3%	0	0.0%	0	0.0%	2	10.0%
Failure of Septoplasty	4	4.7%	0	0.0%	4	22.2%	0	0.0%
No Answer	6	7.0%	2	4.2%	0	0.0%	4	20.0%

**Table 5:** Accuracy of physical exam to determine who requires surgery

	All Physicians		General Otolaryngology		FPRS		Rhinology	
	n	%	n	%	n	%	n	%
Low	14	16.3%	8	16.7%	4	22.2%	2	10.0%
Moderate	48	55.8%	28	58.3%	6	33.3%	14	70.0%
High	24	27.9%	12	25.0%	8	44.4%	4	20.0%

*p = 0.23

### Management

All respondents performed septoplasties, with a mean (standard deviation) of 71.2 (60.7) surgeries per year (Table 6). Similarly, 74.4% of respondents performed nasal valve surgeries, with a mean of 18.4(17.2) surgeries per year (Table 6). Significantly more Rhinologists and FPRS perform nasal valve surgery (*p* < 0.001), and FPRS perform significantly more yearly procedures (*p* = 0.004). Respondents indicated that 25.4% of patients with symptomatic septal deviation had comorbid NVC (Table 6). Interestingly, 79.1% of surgeons indicated that less than 50% of patients with evidence of NVC and septal deviation required a functional rhinoplasty in addition to septoplasty, while 9.3% of surgeons indicated all patients with evidence of NVC required a functional rhinoplasty (Table 7). There was a significant difference of opinions between subspecialties regarding percentage of patients who require nasal valve surgery (*p* = 0.001, Table 7). In addition, statistically significant differences were found between FPRS and other surgeons in terms of the percentage of patients who require a functional rhinoplasty (*p* = 0.001, Table 6).

**Table 6:** Stratifying by speciality

Variable	TOTAL		General		FPRCS		Rhinology		
	Mean	SD	Mean	SD	Mean	SD	Mean	SD	*P*-value
Surgeons who perform Septoplasties	86		48		18		20		> 0.9
Physicians Performing Surgery of the Nasal valve	64		28		18		18		0.33
Number of Septoplasties / year	71.2	60.7	54.4	43.9	109.4	81.8	46.3	59.5	0.004
Number of nasal valve surgeries /year	18.4	17.2	13.1	13.1	32.4	21.9	13.3	10.6	0.004
Percentage of Patients with a SD who also have nasal valve collapse	25.4	17.8	25.6	25.6	26.7	16.8	23.8	13.1	0.625
Percentage of patients with a SD and NVC requiring a functional rhinoplasty	35.3	29.7	31.8	31.8	45.6	39.2	33.5	28.1	0.844
Failure Rate of Septoplasty in patient with co-morbid Valve Collapse (no valve surgery performed)	33.7	24.8	34.7	34.7	44.1	26.3	17.8	11.0	0.012

**Table 7:** % with symptomatic septal deviation and NVC who require nasal valve surgery

	TOTAL		General		FPRCS		Rhinology	
	n	%	n	%	n	%	n	%
None	6	7.0	6	12.5	0	0.0	0	0
< 50%	62	72.1	34	70.8	8	44.4	20	100
> 50%	10	11.6	4	8.3	6	33.3	0	0
All	8	9.3	4	8.3	4	22.2	0	0

*p = 0.001

### Prognosis

According to our survey, the most commonly identified cause of a failed septoplasty was inadequate septoplasty followed by untreated NVC (Table 8). Interestingly, results were similar for all subspecialties apart from 22.2% of FPRS indicating that a caudal septal deviation was one most common cause. Respondents noted that in patients with evidence of septal deviation and NVC, the mean (SD) percentage of patients who do not improve after a septoplasty alone was 33.7 (24.8) %. Failure in this case was defined as the patient not indicating significant improvement in breathing at 3 months follow-up (Table 6). There was also a significant difference between specialities regarding the failure rate of septoplasty alone (*p* = 0.012, Table 6).

**Table 8:** Most Common Cause of Septoplasty Failure

	TOTAL		General		FPRCS		Rhinology	
	n	% of Total	n	% of Total	n	% of Total	n	% of Total
Inadequate Septoplasty	36	41.9	22	45.8	8	44.4	6	30.0
Nasal Valve Collapse	22	25.6	10	20.8	6	33.3	6	30.0
Mucosal Obstruction (Turbinates/Allergies)	6	7.0	4	8.3	0	0.0	2	10.0
Other	6	7.0	4	8.3	0	0.0	2	10.0
Caudal Septal Deviation	6	7.0	0	0.0	4	22.2	0	0.0
Maxillary Crest Spur	4	4.7	2	4.2	0	0.0	2	10.0
Unrealistic Expectations	2	2.3	3	6.3	0	0.0	0	0.0
Recurrance of Deviation	2	2.3	1	2.1	0	0.0	2	10.0
Synechia	2	2.3	2	4.2	0	0.0	0	0.0

## Discussion

This survey represents the first survey of Canadian Otolaryngologists assessing their method of diagnosis and treatment of both septal deviation as well as nasal valve collapse. Respondents in our study identified a wide variety of diagnostic methods, with the assumption that multiple tests are utilized to come to a diagnostic conclusion. Although we attempted to determine what examination physicians felt was most important, it is unclear from our survey what the relative weight each physician applies for each test when there is a discrepancy between tests. Research assessing the relative efficacy of each test as well as the combined effect of tests may assist surgeons in making evidence based decisions.

Our survey demonstrated that the Cottle Manoeuvre is both a common and important tool for NVC diagnosis. These findings are consistent with a recent systematic review, which demonstrated that the Cottle Manoeuvre was the most common method used to determine whether a patient required surgical repair [12]. This manoeuvre however, has been described in literature as non-specific, as many patients without NVC will also feel an improvement in airway patency [13, 14]. Furthermore, false negatives can occur such as in the case of osteum internum fibrosis [15]. A recent study demonstrated no change in outcome in surgical success after a septoplasty in patients with either positive or negative Cottle Maneouver [16]. These results put into question the utility of the notion that patients with a positive Cottle Maneouver, when used as a single examination, truly benefit from anything more than a septoplasty.

Interestingly, there were differences in opinions between subspecialties. FPRS indicated a higher failure rate of septoplasty alone, as well as a higher percentage of patients who require nasal valve surgery than other subspecialties. There may be multiple reasons for this discrepancy. First, the indication for referral to subspecialists may vary, such as nasal polyps referred to Rhinology versus nasal trauma referred to FPRS. Furthermore, more complicated cases of NVC may be referred to FPRS from other otolaryngologists, and both patient and physician may be more open to surgery as a final option. FPRS also perform more nasal valve surgeries, which may due to a higher number of referrals for NVC than other otolaryngologists. It is also important to note that our results may also suggest a difference in protocol for diagnosis and management of NVC between specialists.

There were some limitations to our study. The first is the response rate to our survey (Table 1). 18% of otolaryngologists responded from CSO, which is below the recommended guidelines of 60% to minimize nonresponse bias [17]. The CSOHNS includes many surgeons who may not perform septorhinoplasties in adults (Pediatrics, Otology, Laryngology, Head and Neck), thus our response rate is likely an underestimation of the true rate if only interested surgeons were included. External validity was also a challenge, as only 3 subspecialists replied to our survey. It is likely that survey results would be different for other subspecialists who have less experience using certain manoeuvres described in the survey. Furthermore, the majority of respondents in our study had less than 10 years of experience, which may have an impact on which diagnostic tests were performed.

The results of this study highlight the heterogeneity in terms of diagnosing valve collapse as well as the opinions on treatment indications and methods. The results of this study will assist in developing guidelines for the diagnosis of nasal valve collapse.

Future studies should aim to assess the effectiveness of diagnostic tests for NVC. If septal deviation and NVC can be accurately diagnosed, management could be optimized and prognosis may potentially improve. Interestingly, there were no responders who identified the Latera Implant as a potential treatment option when given the opportunity in open ended questions in the survey. The Latera is a novel treatment for NVC inserted in the office or the operating room [18]. This is a relatively new treatment and likely was not considered by those who responded to the study. It is unclear if prompted as a choice in our survey, would more surgeons have indicated their use of this treatment.

Furthermore, as anatomy does not always correlate with obstruction for septal deviation and NVC, it may be important to determine novel outcome measures to assist in management [19]. The NOSE score is one outcome measure that may be used for this particular purpose [16]. In addition, it is unknown if certain tests are applicable to both types of collapse, and more studies are needed to evaluate methods to differentiate internal and external NVC. For differentiation, one study suggested that cotton in the angle of the internal nasal valve simulating spreader grafts could be used [20]. Furthermore, although the Modified Cottle Maneouver was never explicitly discussed in literature for this particular purpose, from experience it may also be used to determine location of collapse.

## Conclusions

Our findings indicate NVC is an important concern for otolaryngologists performing septoplasty. Although most physicians indicate a moderate effectiveness of the physical exam, diagnostic procedure for NVC is variable. The Cottle Maneouver is often relied on for external NVC, however its effectiveness has been challenged. Stratifying by speciality, FPRS note a higher failure rate of septoplasty alone, and believe more patients require NVC surgery than other specialists.

## Data Availability

Datasets are attached as an additional file.

## Abbreviations

AAO-HNS: American Academy of Otolaryngology – Head and Neck Surgery
CSOHNS: Canadian Society of Otolaryngology – Head and Neck Surgery
FPRS: Facial Plastics and Reconstruction Surgeons
NVC: Nasal Valve Collapse
